# Striatal TRPV1 activation by acetaminophen ameliorates dopamine D_2_ receptor antagonist–induced orofacial dyskinesia

**DOI:** 10.1172/jci.insight.145632

**Published:** 2021-05-24

**Authors:** Koki Nagaoka, Takuya Nagashima, Nozomi Asaoka, Hiroki Yamamoto, Chihiro Toda, Gen Kayanuma, Soni Siswanto, Yasuhiro Funahashi, Keisuke Kuroda, Kozo Kaibuchi, Yasuo Mori, Kazuki Nagayasu, Hisashi Shirakawa, Shuji Kaneko

**Affiliations:** 1 Department of Molecular Pharmacology, Graduate School and Faculty of Pharmaceutical Sciences, Kyoto University, Kyoto, Japan.; 2 Department of Pharmacology, Kyoto Prefectural University of Medicine, Kyoto, Japan.; 3 Department of Cell Pharmacology, Graduate School of Medicine, Nagoya University, Nagoya, Japan.; 4 Research project for neural and tumor signaling, Institute for Comprehensive Medical Science, Fujita Health University, Toyoake, Japan.; 5 Department of Synthetic Chemistry and Biological Chemistry, Graduate School of Engineering and Faculty of Engineering, Kyoto University, Katsura Campus, Kyoto, Japan.

**Keywords:** Neuroscience, Drug therapy

## Abstract

Antipsychotics often cause tardive dyskinesia, an adverse symptom of involuntary hyperkinetic movements. Analysis of the US Food and Drug Administration Adverse Event Reporting System and JMDC insurance claims revealed that acetaminophen prevented the dyskinesia induced by dopamine D_2_ receptor antagonists. In vivo experiments further showed that a 21-day treatment with haloperidol increased the number of vacuous chewing movements (VCMs) in rats, an effect that was inhibited by oral acetaminophen treatment or intracerebroventricular injection of *N*-(4-hydroxyphenyl)-arachidonylamide (AM404), an acetaminophen metabolite that acts as an activator of the transient receptor potential vanilloid 1 (TRPV1). In mice, haloperidol-induced VCMs were also mitigated by treatment with AM404 applied to the dorsal striatum, an effect not seen in TRPV1-deficient mice. Acetaminophen prevented the haloperidol-induced decrease in the number of c-Fos^+^preproenkephalin^+^ striatal neurons in wild-type mice but not in TRPV1-deficient mice. Finally, chemogenetic stimulation of indirect pathway medium spiny neurons in the dorsal striatum decreased haloperidol-induced VCMs. These results suggest that acetaminophen activates the indirect pathway neurons by activating TRPV1 channels via AM404.

## Graphical abstract

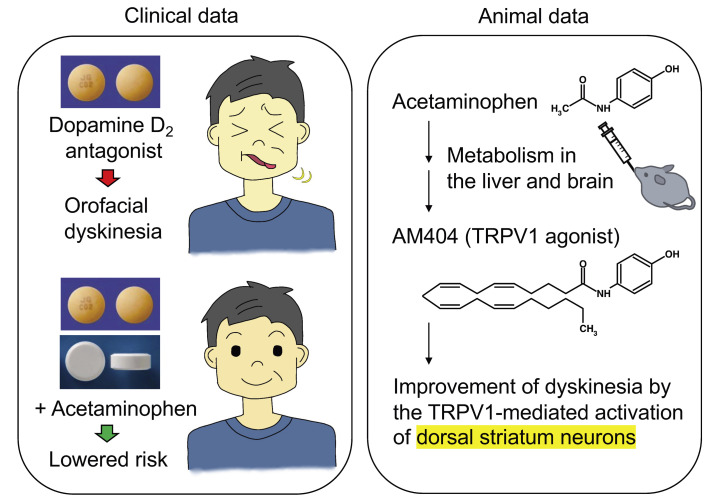

## Introduction

Dyskinesia is a neurological symptom characterized by involuntary muscle movements of the tongue, lower face, jaw, and/or extremities. There are 2 types of drug-induced dyskinesia that result from different pathogenic situations: the levodopa-induced dyskinesia that occurs in patients with Parkinson’s disease and tardive dyskinesia (TD) that occurs due to long-term use of dopamine D_2_ receptor (D2R) antagonists, such as antipsychotics. Dyskinesia also occurs spontaneously as a hyperkinetic symptom of Huntington’s disease and is thus considered related to the degeneration or malfunction of indirect pathway medium spiny neurons (iMSNs) within the striatum (1).

TD emerges after prolonged use of antipsychotics in 20% to 30% of cases, and its symptoms become irreversible after long-term use of D2R antagonists (2). Various clinical treatments using cholinergic agents, such as amantadine, β-adrenergic blockers, GABA agonists, or antioxidants have been explored as therapeutic strategies to prevent or alleviate TD (3); however, there is insufficient evidence to support their use in the management of TD. Recently, specific inhibitors of the vesicular monoamine transporter 2 (VMAT2) have been approved for the treatment of TD by the US Food and Drug Administration (FDA); however, according to a recent warning (4), the use of a VMAT2 inhibitor might cause depression and suicidal ideation by depleting the brain monoamines. Thus, the development of new strategies to reduce the risk of TD remains a priority.

One approach for finding effective treatments for drug-induced adverse events such as TD is to analyze clinical big data and search for hidden drug-drug interactions. The FDA Adverse Event Reporting System (FAERS) is the world’s largest freely available database of self-reported adverse events. Several novel, unexpected drug-drug interactions have been identified by analyzing the FAERS (5, 6). In these studies, the incidence of adverse events of a drug of interest (drug A) was markedly inhibited by the concomitant use of another drug (drug B), which was validated by electronic health records or animal experiments. Therefore, analyses of FAERS data may represent a valuable strategy to generate new hypotheses on the confounding factors of a particular clinical adverse event. However, there is a methodological drawback arising from the lack of a causal relationship between the concomitant use of drug B and changes in the incidence of adverse events in the FAERS. To address this issue, we coupled FAERS data analysis with information collected from JMDC insurance claims database (formerly known as the Japan Medical Data Center), which contains the monthly medical records of diagnoses, treatments, and prescriptions of 5.5 million corporate employees and their dependent family members, to investigate the chronological sequence of drug-induced dyskinesia.

We first explored the FAERS and JMDC data to determine a negative confounder drug for the occurrence of drug-induced dyskinesia and found that acetaminophen could represent a promising candidate for the treatment of dyskinesia. We then validated its efficacy in rodent models to determine its underlying therapeutic targets and mechanisms.

## Results

### Drug-induced dyskinesia in FAERS data.

**Figure 1:**
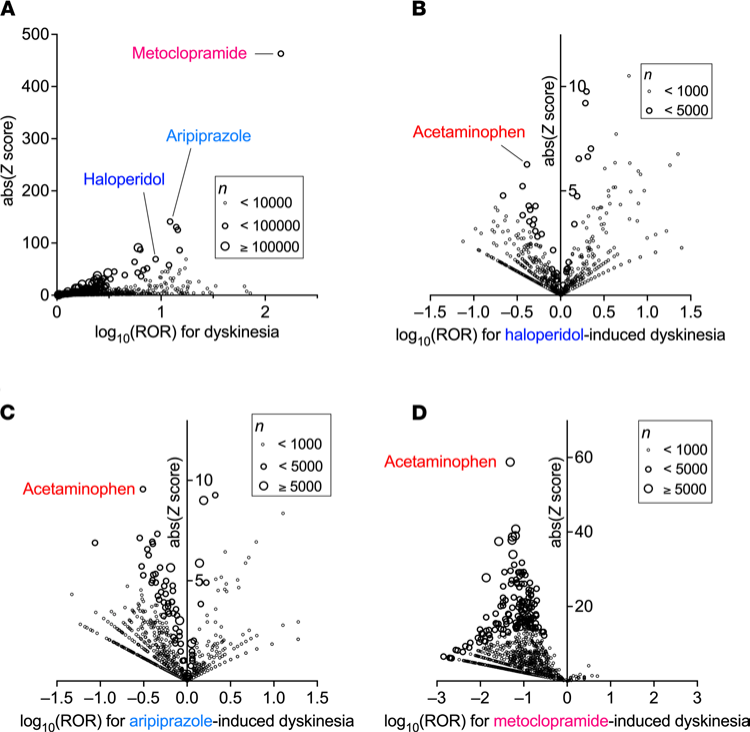
Increased ROR of dyskinesia with the use of dopamine D2R antagonists and confounding effects of concomitant drugs on the drug-induced dyskinesia in FAERS data. Statistical analyses of FAERS data (*n* = 9,948,368 from 2004 to 2018) were performed using R v4.0.2 and R studio v1.3.959 software. Precise equations used in these analyses, all the drugs, their ROR values, and *z* scores are shown in Supplemental Tables 1 and 2. Volcano plots of ROR in a log scale and the statistical significance (absolute *z* score) are shown. Each circle indicates an individual drug, and the size of the circle reflects the number of patients taking the drug. (**A**) Strong and significant increases in the ROR of dyskinesia were seen in patients using D2R antagonists, such as metoclopramide, aripiprazole, and haloperidol. (**B**–**D**) Within the population taking each one of the D2R antagonists, the confounding effects of concomitantly used drugs on the ROR of drug-induced dyskinesia are shown.

First, we investigated the association between the use of drugs and the incidence of dyskinesia in FAERS data via disproportionality analysis by calculating the reporting odds ratio (ROR) and its *z* score. Due to the known reporting bias and the lack of incidence denominators accompanied by self-reports, these values do not reflect the real incidence rate (7). Nevertheless, a group of more than 10 D2R antagonists exhibited a strong association between their use and the emergence of dyskinesia with higher ROR and *z* scores (Figure 1A and Supplemental Table 1; supplemental material available online with this article; https://doi.org/10.1172/jci.insight.145632DS1). Hence, we chose 3 D2R antagonists of different pharmacological categories for further analysis: the typical antipsychotic haloperidol, the atypical antipsychotic aripiprazole with D2R partial agonist properties, and the antiemetic metoclopramide. The number of cases was large enough to further investigate potential confounders of drug-induced dyskinesia. Associated with these antipsychotics, dyskinesia is the most frequent adverse event with the highest ROR values. We did not select quetiapine, olanzapine, or other atypical antipsychotics because these agents have strong metabolic adverse effects that may hinder and underestimate ROR values for dyskinesia.

### Confounding effect of acetaminophen on the ROR of D2R antagonist–induced dyskinesia.

When the confounding effects of all the drug combinations on the ROR of dyskinesia were evaluated in populations using each of the D2R antagonists, many concomitantly used drugs affected the ROR of drug-induced dyskinesia (Figure 1, B–D) without changing the ROR of dyskinesia by themselves. Among the drugs that lowered the ROR value, acetaminophen showed the highest absolute *z* score (Supplemental Table 2). Other candidate drugs with strong mitigating effects included aspirin, proton pump inhibitors, thyroxine, diuretics, and central nervous system (CNS) depressants.

### Incidence rate of dyskinesia after the use of D2R antagonists in JMDC insurance claims data.

To investigate the correlation between D2R antagonist use and clinical dyskinesia, we analyzed the JMDC insurance claims data. Looking at the time distribution of the first event after enrollment in the JMDC (Supplemental Figure 1), the number of patients initially diagnosed with dyskinesia and prescribed haloperidol or aripiprazole was much higher during the first 2 months and became stable after 3 months, whereas that of patients with dyskinesia and prescribed metoclopramide was also higher until 2 months but exhibited a circannual pattern, suggesting an increased concomitant use of metoclopramide during the winter with nonsteroidal antiinflammatory drugs (as in Japan, most patients are enrolled in April, when the academic and financial year starts). These results suggest that patients who received a diagnosis of dyskinesia or were prescribed haloperidol, aripiprazole, or metoclopramide within 2 months after enrollment may exhibit dyskinesia before or just after enrollment. Therefore, these patients (who received a diagnosis of dyskinesia or prescription of haloperidol, aripiprazole, or metoclopramide during the 0- to 2-month run-in period) were removed from the study cohort.

Next, we evaluated the overall association between the use of D2R antagonists and the onset of dyskinesia by estimating the incidence rate ratio (IRR) of dyskinesia. Haloperidol and aripiprazole showed high IRR values, whereas the increase by metoclopramide was small but significant in the JMDC cohort (Supplemental Table 3), probably reflecting the potency of the D2R antagonists.

When extracting the 3 populations that received haloperidol, aripiprazole, or metoclopramide, we performed 1:1 propensity score matching (Supplemental Table 4) to eliminate known confounding factors for dyskinesia, such as older age, female sex, antiparkinsonian drug use, additional antipsychotic use, alcohol and substance abuse/dependence, and diagnosis of mood disorders, diabetes mellitus, or hepatic diseases (3). In these matched cohorts taking D2R antagonists, daily and cumulative doses, and the administration period of D2R antagonists, were equivalent in each pair with or without acetaminophen (Supplemental Table 5). However, the profile of these cohorts suggested that the haloperidol and metoclopramide cohort pairs were not suitable for further analysis of dyskinesia. In the haloperidol cohort, only 11 patients exhibited dyskinesia within a median of 2 months of haloperidol administration, with a cumulative dose of 80 mg, which is not a sufficient number of cases, with a small dose and a short period of use. In the metoclopramide cohort, the median administration period was only 4 to 5 days, with a cumulative dose of 60 to 70 mg, which was also insufficient to be considered as causative of TD. In contrast, since the aripiprazole cohort pair showed an appropriate cumulative dose (median more than 300 mg) and administration period (more than 3 months) with a sufficient number of dyskinesia cases, we focused on this cohort pair for further analysis.

### Concomitant use of acetaminophen alleviates antipsychotic-induced dyskinesia in JMDC data.

**Figure 2:**
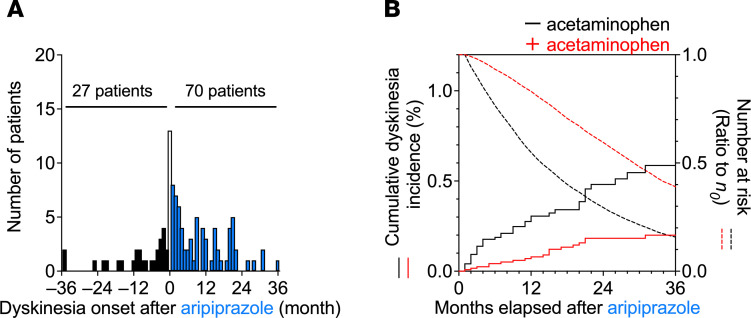
Time trends in the incidence of dyskinesia in the cohorts prescribed aripiprazole among the JMDC claims data. Patients who had been enrolled in the health insurance for 0–2 months were omitted for the analysis to allow a run-in period (based on the results indicated in Supplemental Figure 1), and the population characteristics were matched as shown in Supplemental Table 4. (**A**) Sequence symmetry analysis showing the causal relationship with an adjusted sequence ratio of 3.3 (95% CI: 2.2–5.4) between the start of aripiprazole treatment and the onset of dyskinesia in an observation period of ±36 months (*n* = 97). At month 0 (white bar), the precise chronological order was unclear, and the data were omitted from the analysis. (**B**) Kaplan-Meier curves for the cumulative incidence ratio of dyskinesia in patients taking aripiprazole are shown individually in 2 groups: without (black) and with (red) coprescribed acetaminophen. Cox proportional hazards modeling indicated that the combination with acetaminophen significantly decreased the aripiprazole-induced incidence of dyskinesia, with a hazard ratio of 0.33 (95% CI: 0.19–0.58, *P* = 4.0 × 10^−5^ in the log-rank test). The dotted lines show the number of patients at risk, as a ratio to the initial number of patients (*n*_0_ = 12,216 for both groups).

In the aripiprazole cohort, a significant causal association between aripiprazole use and dyskinesia onset was detected with an adjusted sequence ratio of 3.3 (95% CI: 2.2–5.4) in sequence symmetry analysis of the propensity score–matched cohorts (Figure 2A). Kaplan-Meier analysis and Cox proportional hazards modeling indicated that the combination with acetaminophen significantly decreased the aripiprazole-induced incidence of dyskinesia with a hazard ratio of 0.33 (95% CI: 0.19–0.58, *P* = 4.0 × 10^−5^ in the log-rank test, Figure 2B). In parallel, the decay in the number of remaining patients at risk was also attenuated by acetaminophen in the aripiprazole cohorts, suggesting that dropout from long-term aripiprazole treatment was avoided by acetaminophen. These real-world patient data demonstrate that the concomitant use of acetaminophen alleviates antipsychotic-induced dyskinesia.

### Acetaminophen inhibits haloperidol-induced increases in orofacial dyskinetic symptoms in rats.

To determine whether acetaminophen could mitigate D2R antagonist–induced dyskinesia in vivo, we used a vacuous chewing movement (VCM) model of TD induced by repetitive treatment of rodents with haloperidol. Aripiprazole was not used because haloperidol is the only agent that reliably elicits long-lasting orofacial dyskinesia in rodents (8). Although long-term treatment with haloperidol is preferable to establish a TD-like syndrome, we chose a short-term protocol based on 3 weeks of treatment that caused sufficient behavioral and biochemical changes in rats (9). In the present study, we measured the number of VCMs 24 hours after the last haloperidol treatment. A marked increase in the number of VCMs was observed at 24 hours after daily haloperidol was administered to rats (1 mg/kg/d) and mice (2 mg/kg/d) for 21 days. Moreover, the VCM behavior lasted for at least 3 days during the withdrawal phase of haloperidol (Supplemental Figure 2).

**Figure 3:**
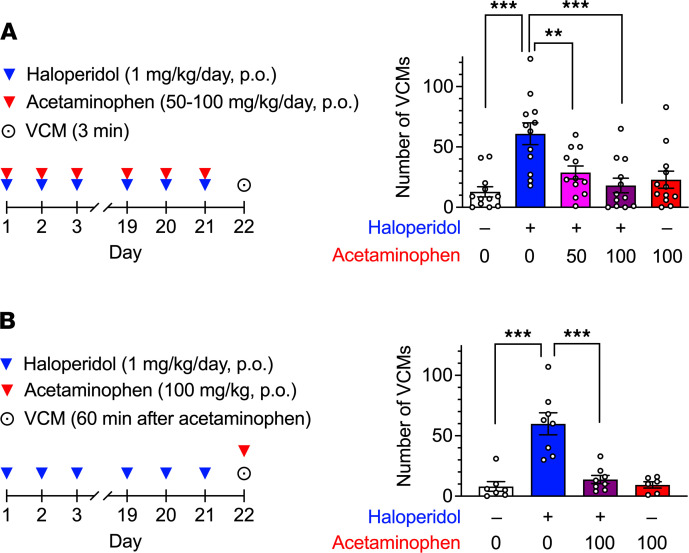
Effects of acetaminophen on haloperidol-induced VCMs in rats. (**A**) Rats (*n* = 12 per group) were treated daily with haloperidol (1 mg/kg/d), acetaminophen (50 or 100 mg/kg/d), or both, administrated orally for 21 days, and the number of VCMs was measured for 3 minutes starting 24 hours after the last administration. (**B**) Rats (*n* = 6–8 per group) were treated daily with oral haloperidol (1 mg/kg/d) for 21 days and then received oral acetaminophen (100 mg/kg) at 23 hours after the last administration of haloperidol. The number of VCMs was measured for 3 minutes starting 60 minutes after acetaminophen or vehicle administration. Statistical significance was tested using 1-way ANOVA with a post hoc Tukey’s test. **P < 0.01; ***P < 0.001.

When rats were cotreated with haloperidol (1 mg/kg/d, orally [po]) or acetaminophen (50 or 100 mg/kg/d, po), the number of VCMs was significantly decreased compared with that of rats treated with haloperidol alone (Figure 3A, 1-way ANOVA, *F*_4,55_ = 8.37, *P* < 0.001). Moreover, when acetaminophen (100 mg/kg, po) was administered to rats the day after 21 days of treatment with haloperidol (1 mg/kg/d, po), the number of VCMs was also significantly decreased (Figure 3B, 1-way ANOVA, *F*_3,25_ = 19.2, *P* < 0.001). These results suggest that acetaminophen is effective in preventing the development of dyskinesia and acutely inhibiting orofacial symptoms.

When the impact of acetaminophen on the antipsychotic effect of haloperidol was tested, the hyperlocomotion induced by methamphetamine was found to be significantly suppressed by haloperidol, whereas it was not affected by acute treatment with 100 mg/kg acetaminophen (Supplemental Figure 3A, 1-way ANOVA, *F*_3,12_ = 24.8, *P* < 0.001). Moreover, no significant change in the total distance traveled in 30 minutes by the rats that received haloperidol, acetaminophen, or both for 21 days was observed (Supplemental Figure 3B, 1-way ANOVA, *F*_4,35_ = 0.43, *P* = 0.78). Additionally, there was no difference in the serum levels of aspartate aminotransferase (1-way ANOVA, *F*_4,15_ = 1.09, *P* = 0.40), alanine aminotransferase (*F*_4,15_ = 1.28, *P* = 0.32), albumin (*F*_4,15_ = 0.36, *P* = 0.83), and creatinine (*F*_4,15_ = 1.72, *P* = 0.20) in rats treated with haloperidol, acetaminophen, or both for 21 days (Supplemental Figure 3, C–F); however, acetaminophen overdose is known to induce hepatopathy and nephropathy in humans (10, 11).

### Intracerebral administration of an acetaminophen metabolite mimics the inhibitory effect of acetaminophen on haloperidol-induced dyskinesia in rodents.

**Figure 4:**
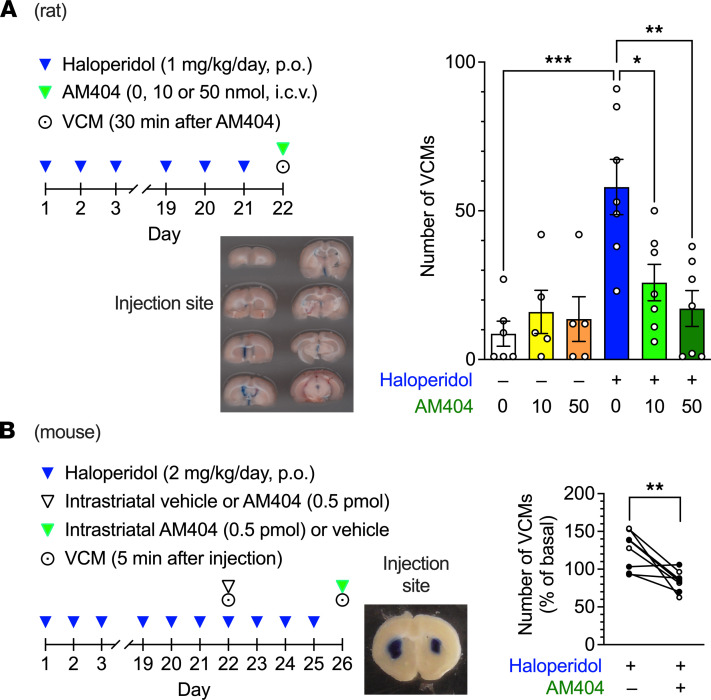
Effects of intracerebral AM404 on haloperidol-induced VCMs in rodents. (**A**) Rats (*n* = 5–7 per group) were treated daily with oral haloperidol (1 mg/kg/d) for 21 days and received AM404 (0, 10, or 50 nmol) via intracerebroventricular injection at 23 hours after the last administration of haloperidol. The number of VCMs was measured for 3 minutes starting 30 minutes after the administration of AM404. After the experiment, the injection sites were confirmed by injecting Evans blue through the same cannula. A representative image of consecutive coronal sections is shown (43 mm wide in actual size). Individual data are shown with the mean ± SEM. Statistical significance was tested using 1-way ANOVA with post hoc Tukey’s test. *P < 0.05, **P < 0.01, ***P < 0.001. (**B**) Mice (*n* = 8 per group) were treated daily with oral haloperidol (2 mg/kg/d) for 21 days. On day 22, after VCMs were counted for 5 minutes as an initial baseline, half of the mice received vehicle (group 1), and the rest received AM404 (0.5 pmol/side; group 2) through a preimplanted cannula in the dorsal striatum. After 5 minutes, the number of VCMs was counted for another 5 minutes to determine the effect of the injected substance. The daily haloperidol treatment was continued for 4 more days; on day 26, the second set of VCM measurements was performed in a crossover design, with AM404 injection in group 1 and vehicle injection in group 2. After the experiment, the injection sites were confirmed by Evans blue staining through the same cannula. A representative image of the coronal section is shown (11.5 mm wide in actual size). Changes in the number of VCMs are represented for individual mice on days 22 and 26 as a percentage of the baseline VCM count. Open and filled circles indicate the results from groups 1 and 2, respectively. Statistical significance was tested using 2-tailed paired *t* test. **P < 0.01.

Acetaminophen is metabolized in the CNS to *p*-aminophenol and converted to *N*-(4-hydroxyphenyl)-arachidonylamide (AM404) via conjugation with arachidonic acid catalyzed by fatty acid amide hydrolase (FAAH, ref. 12). To test the possibility that the antidyskinetic effect of acetaminophen was mediated by activity of its metabolite AM404, we injected AM404 into the intracerebroventricular space of rats via a preimplanted cannula after 21 days of treatment with haloperidol. When the number of VCMs was measured 30 minutes after an intracerebroventricular injection of AM404 (10 or 50 nmol), the frequency of haloperidol-induced VCM events decreased in a dose-dependent manner (Figure 4A, 1-way ANOVA, *F*_5,31_ = 7.03, *P* < 0.001), suggesting the involvement of AM404 in the inhibitory effect of acetaminophen on orofacial dyskinesia. However, the dose of AM404 required to decrease the number of VCMs was higher than expected. As revealed by the distribution of Evans blue dye in consecutive coronal sections, AM404 might not diffuse sufficiently to the parenchymal brain tissue of rats after a single intracerebroventricular injection. Considering that iMSNs within the dorsal striatum are involved in dyskinesia (1), we used a haloperidol-induced VCM mouse model and applied AM404 directly to their dorsal striatum. In a crossover test in which the number of VCMs was repeatedly measured twice on days 22 and 26 before, and 5 minutes after, the infusion of vehicle or AM404, a significant decrease in the relative number of VCMs after a bilateral dorsal striatal infusion of AM404 (0.5 pmol/side) was observed when compared with the vehicle infusion control (Figure 4B, paired *t* test, *P* < 0.01).

### Antidyskinetic effect of acetaminophen is not observed in transient receptor potential vanilloid 1–KO mice.

Transient receptor potential vanilloid 1 (TRPV1) channels are expressed in peripheral sensory neurons and in the CNS (13). The antinociceptive effect of acetaminophen is mediated by the activation of central TRPV1 channels via AM404 (14), whereas the hypothermic effect of acetaminophen is mediated by transient receptor potential ankyrin 1 (TRPA1) channels (15) in the CNS. To clarify the involvement of TRPV1 and TRPA1 in the antidyskinetic effect of acetaminophen, we investigated the effects of acetaminophen on haloperidol-induced VCMs using B6.129X1-Trpv1^tm1Jul^/J, (TRPV1-KO) and B6.129P-Trpa1^tm1Kykw^/J (TRPA1-KO) mice and also monitored the acetaminophen-induced changes in body temperature.

**Figure 5:**
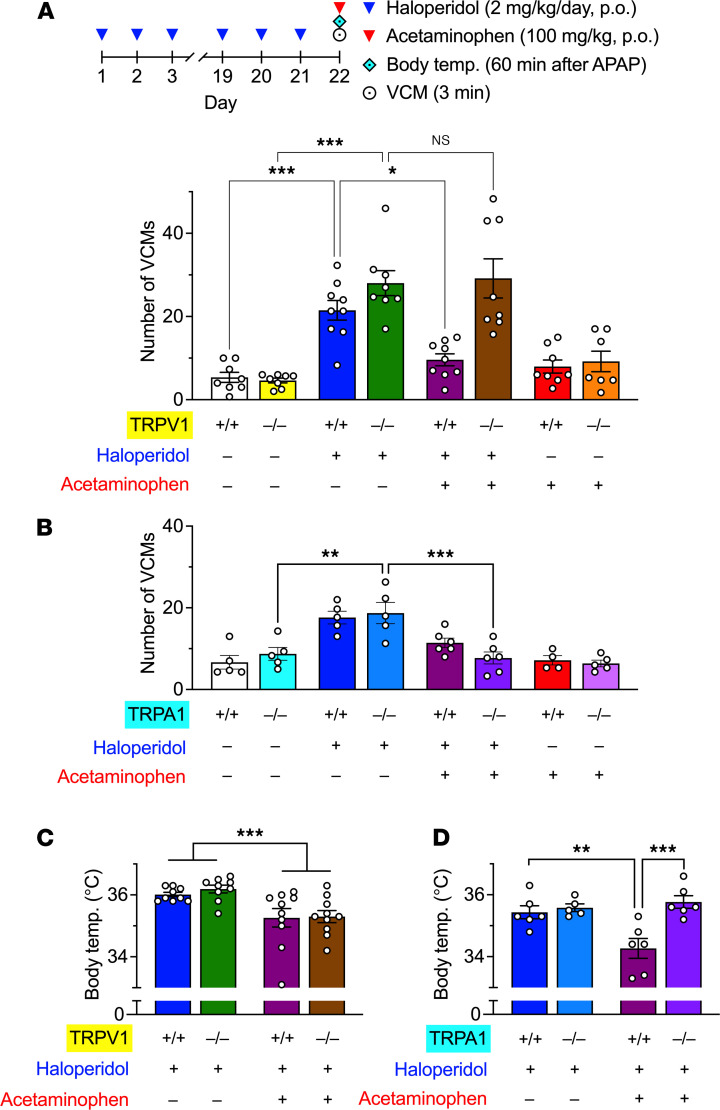
Effects of acute acetaminophen on haloperidol-induced VCMs and body temperature in WT, TRPV1-KO, and TRPA1-KO mice. (**A** and **B**) WT, TRPV1-KO, and TRPA1-KO mice (*n* = 4–9 per group) were treated daily with orally administrated haloperidol (2 mg/kg/d) for 21 days; 23 hours after the last administration, mice received oral acetaminophen (300 mg/kg) or vehicle. The number of VCMs was counted for 3 minutes beginning 60 minutes after the last administration. APAP, acetaminophen. (**C** and **D**) WT, TRPV1-KO, and TRPA1-KO mice (*n* = 5–10 per group) were treated daily with orally administrated haloperidol (2 mg/kg/d) for 21 days. At 23 hours after the last administration of haloperidol, the mice were treated with oral acetaminophen (300 mg/kg) or vehicle, and their rectal temperature was measured 60 minutes later. Individual data are shown as mean ± SEM. Statistical significance was tested using 2-way ANOVA with post hoc multiple comparisons; *P < 0.05; **P < 0.01; and ***P < 0.001; NS, not significant.

In mice treated daily with haloperidol (2 mg/kg/d, po) for 21 days, a significant increase in the number of VCMs was observed in both wild-type (WT) and TRPV1-KO mice on day 22. Acute treatment with acetaminophen (300 mg/kg, po) significantly inhibited haloperidol-induced VCMs in WT but not in TRPV1-KO mice (Figure 5A, 2-way ANOVA, drug: *F*_3,57_ = 28.3, *P* < 0.001, genotype: *F*_1,57_ = 14.6, *P* < 0.001; interaction: *F*_3,57_ = 7.05, *P* < 0.001). In contrast, there was no difference in the effects of haloperidol and acetaminophen between WT and TRPA1-KO mice (Figure 5B, 2-way ANOVA, drug: *F*_3,33_ = 20.9, *P* < 0.001; genotype: *F*_1,33_ = 0.09, *P* = 0.76; interaction: *F*_3,33_ = 1.42, *P* = 0.26), indicating that acetaminophen exhibits an antidyskinetic effect via TRPV1 but not TRPA1 channels.

When the body temperature was measured in the same schedule as the VCMs, significant hypothermia was caused by acetaminophen in both WT and TRPV1-KO mice (Figure 5C, 2-way ANOVA, drug: *F*_1,34_ = 16.8, *P* < 0.001, genotype: *F*_1,34_ = 0.30, *P* = 0.59, interaction: *F*_1,34_ = 0.12, *P* = 0.73), whereas this was not observed in TRPA1-KO mice (Figure 5D, 2-way ANOVA, drug: *F*_1,19_ = 12.4, *P* < 0.01; genotype: *F*_1,19_ = 4.38, *P* < 0.05; interaction: *F*_1,19_ = 8.36, *P* < 0.01), suggesting that the hypothermic effect of acetaminophen is mediated via TRPA1 channels.

### Intrastriatal injection of AM404 or capsaicin inhibits haloperidol-induced VCMs via TRPV1.

**Figure 6:**
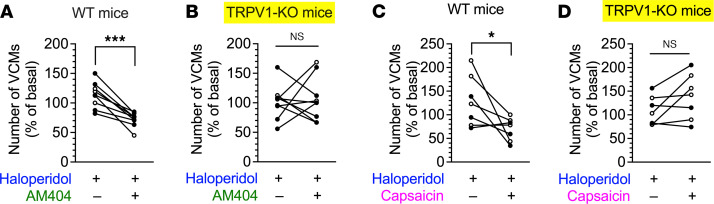
Effects of the dorsal striatal injection of AM404 and capsaicin on haloperidol-induced VCMs in WT and TRPV1-KO mice. WT and TRPV1-KO mice were treated daily with orally administrated haloperidol (2 mg/kg/d) for 21 days. On day 22, after VCMs were counted for 5 minutes as an initial baseline, half of the mice received vehicle (group 1), and the rest received AM404 (1 pmol/side) or capsaicin (10 ng/side) (group 2) through a preimplanted cannula in the dorsal striatum. Five minutes later, the number of VCMs was counted for another 5 minutes to determine the effect of the injected drug. The daily haloperidol treatment was continued for 4 more days; on day 26, the second set of VCM measurements was performed in a crossover design, with AM404 or capsaicin injection in group 1 and vehicle injection in group 2. (**A** and **B**) Effects of the dorsal striatal injection of AM404 on haloperidol-induced VCMs (*n* = 9 per group). (**C** and **D**) Effects of the dorsal striatal injection of capsaicin on haloperidol-induced VCMs (*n* = 7 per group). Changes in the number of VCMs are represented for individual mice on days 22 and 26 as percentages of the baseline VCM count. Open and filled circles indicate the results from groups 1 and 2, respectively. Statistical significance was tested using repeated measures 2-way ANOVA with post hoc multiple comparisons; *P < 0.05; ***P < 0.001.

AM404 was originally synthesized as an inhibitor of cellular anandamide uptake at micromolar concentrations, leading to increased levels of endogenous cannabinoids (16). Subsequently, it was found to be a potent TRPV1 agonist equipotent with capsaicin (17) and a biological acetaminophen metabolite with weak cyclooxygenase inhibitor activity (18). To test the specific involvement of TRPV1 in the antidyskinetic effect of AM404, we infused a high dose of AM404 or of the TRPV1 agonist capsaicin into the dorsal striatum in WT and TRPV1-KO mice in a crossover test and compared the number of VCMs. In WT mice, the number of VCMs decreased after bilateral infusion with 1 pmol/side AM404 when compared with the vehicle infusion control, whereas no significant change was observed in the number of VCMs in TRPV1-KO mice after infusion with vehicle or AM404 (Figure 6, A and B; 2-way ANOVA, drug: *F*_1,32_ = 4.35, *P* < 0.05, genotype: *F*_1,32_ = 1.57, *P* = 0.22, interaction: *F*_1,32_ = 6.83, *P* < 0.05). A bilateral intrastriatal infusion with capsaicin (10 ng/side) also suppressed the haloperidol-induced VCMs in WT mice but not in TRPV1-KO mice (Figure 6, C and D; 2-way ANOVA, drug: *F*_1,24_ = 0.91, *P* = 0.35, genotype: *F*_1,24_ = 2.35, *P* = 0.14; interaction: *F*_1,24_ = 8.33, *P* < 0.01).

### Acetaminophen facilitates iMSNs by activating TRPV1.

Striatal GABAergic iMSNs express dopamine D2Rs, adenosine A_2A_ receptors (Adora2a), and enkephalin (19). Previous reports demonstrated that D2R antagonist–induced dyskinesia is accompanied by a decrease in iMSN activity through the hypersensitization of D2Rs (1). Thus, we identified iMSNs in the dorsal striatum with an antipreproenkephalin (ppENK) antibody and evaluated the neural activity of these cells by c-Fos staining.

**Figure 7:**
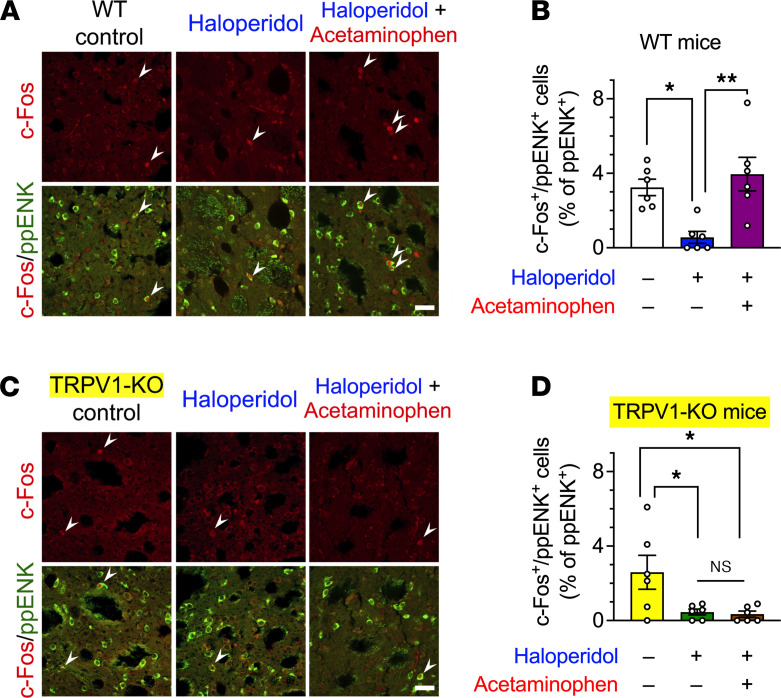
Immunostaining of iMSNs with antibodies against preproenkephalin (ppENK) and c-Fos in WT and TRPV1-KO mice after 21 days of treatment with haloperidol. WT (**A** and **B**) and TRPV1-KO (**C** and **D**) mice (*n* = 6 per group) were treated daily with orally administrated haloperidol (2 mg/kg/d) for 21 days; 23 hours after the last administration of haloperidol, mice were orally treated with acetaminophen (300 mg/kg) or vehicle. After 90 minutes, coronal sections containing the dorsal striatum were prepared, stained with anti–c-Fos and anti-ppENK antibodies, and imaged using confocal microscopy. The numbers of c-Fos^+^ppENK^+^ cells (shown by arrowheads) were counted and are presented as percentages of the number of ppENK^+^ cells, reflecting the total number of iMSNs. Scale bars: 30 μm. Individual data are shown as mean ± SEM. Statistical significance was tested using 1-way ANOVA with a post hoc Tukey’s test. *P < 0.05; **P < 0.01.

In WT mice, c-Fos signals were detected in some of the ppENK^+^ cells (Figure 7A), and the number of c-Fos^+^ppENK^+^ cells was significantly decreased in mice after 21 days of haloperidol (2 mg/kg/d, po) administration, suggesting a reduction in iMSN activity. This decrease was reversed 90 minutes after oral administration of 300 mg/kg acetaminophen (Figure 7B, *F*_2,15_ = 8.64, *P* < 0.01), whereas it failed to improve the number of c-Fos^+^ppENK^+^ cells in haloperidol-treated TRPV1-KO mice (Figure 7, C and D; *F*_2,15_ = 5.46, *P* < 0.05). These results suggest that acetaminophen facilitates the neural activity of iMSNs in the dorsal striatum through the activation of TRPV1 channels.

### Chemogenetic activation of iMSNs prevents haloperidol-induced VCMs.

**Figure 8:**
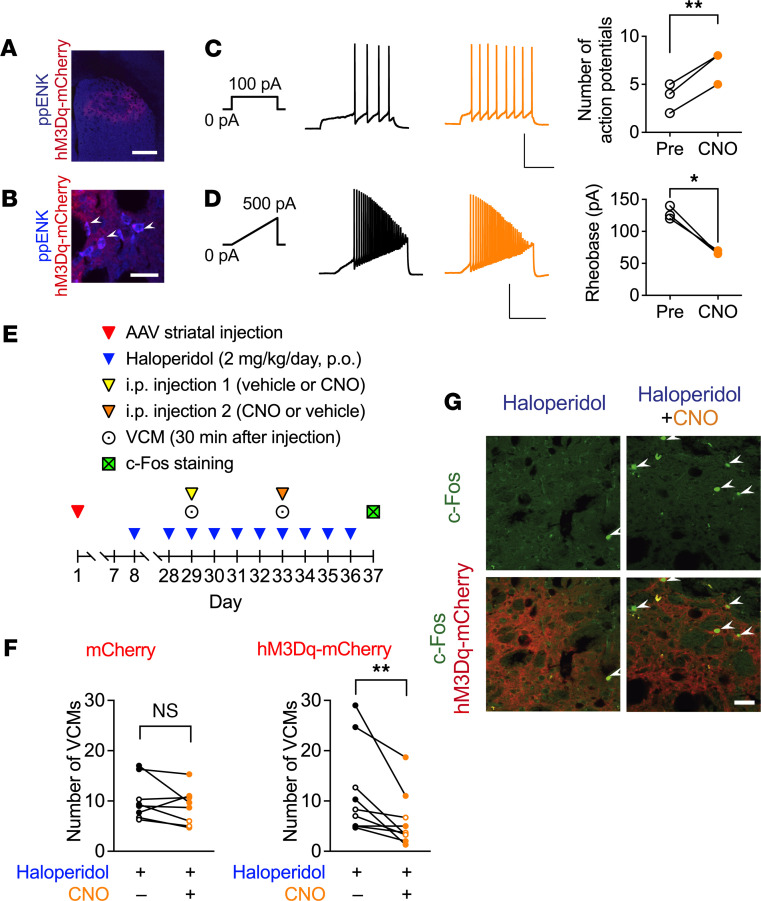
Effects of chemogenetic activation of dorsostriatal neurons on iMSN electrical activity and haloperidol-induced VCMs in mice. Low- (**A**, scale bar: 500 μm) and high-magnification (**B**, scale bar: 30 μm) images indicate coexpression of mCherry fusion protein in ppENK^+^ neurons (shown by arrowheads) throughout the dorsal striatum 4 weeks after the striatal injection of AAV-hSyn-DIO-hM3Dq-mCherry in *Adora2A*-Cre mice. (**C**) Action potentials were evoked by current injection (100 pA, 500 ms) in striatal iMSNs prepared from hM3Dq-mCherry–expressing mice, and the frequency was significantly (*n* = 3, 2-tailed paired *t* test; **P < 0.01) increased after perfusion of the slices with CNO (3 μM). Scale bar: 50 mV, 200 ms. (**D**) Action potentials induced by injecting a ramp current (500 pA, 1 second) before (Pre) and after (CNO) the application of CNO (3 μM) indicate a significant (*n* = 3, 2-tailed paired *t* test; *P < 0.05) decrease in rheobase. Scale bar = 50 mV, 500 ms. (**E**) Experimental protocol for repetitive VCM counting with chemogenetic stimulation of iMSNs. Detailed protocols are documented in the Methods section. (**F**) Changes in the average number of VCMs upon CNO administration are represented for individual mice based on the measurements on days 29 and 33 (*n* = 8–9). Open and filled circles indicate the results from groups 1 and 2, respectively. Statistical significance was tested using repeated measures 2-way ANOVA with post hoc multiple comparisons; **P < 0.01. (**G**) In mice expressing the mCherry tag, an increase was observed in the number of c-Fos^+^ neurons (arrowheads) in coronal striatal sections at 90 minutes after the intraperitoneal administration of CNO (0.5 mg/kg). Scale bar: 30 μm.

To identify whether selective activation of striatal iMSNs is sufficient to suppress haloperidol-induced VCMs, we used transgenic mice expressing Cre recombinase under the control of *Adora2A*-specific promoters and a Cre-inducible adeno-associated viral (AAV) vector encoding the excitatory Gq-coupled human M3 muscarinic receptor (hM3Dq), a designer receptor exclusively activated by designer drugs (20). When the AAV vector was injected into the dorsal striatum of B6.FVB(Cg)-Tg(Adora2a-cre)KG139sat/Mmucd (*Adora2A*-Cre) mice, a fluorescent mCherry signal was observed after 4 weeks throughout the region, and some ppENK^+^ neurons were observed (Figure 8, A and B). When the electrical activity was recorded from striatal slices expressing the mCherry fluorescent tag, iMSNs were identified by the optical morphology and its characteristic slow depolarization and firing pattern in response to current injection (21). The frequency of action potentials increased after perfusion with 3 M clozapine-*N*-oxide (CNO, Figure 8C, paired *t* test: *t*_2_ = 10.0, *P* < 0.01). Analysis of the minimal current amplitude that evoked action potential by applying a ramp current revealed a decrease in the rheobase after CNO administration (Figure 8D, paired *t* test: *t*_2_ = 8.49, *P* < 0.05), reflecting the increased excitability of iMSNs in response to CNO acting on hM3Dq.

Last, haloperidol-induced VCMs were evaluated in *Adora2A*-Cre mice 4 weeks after the intrastriatal injection of the viral vector carrying hM3Dq-mCherry or mCherry alone in a crossover test (Figure 8E). The number of VCMs was not changed by acute CNO treatment in mice that received the control mCherry-expressing vector. However, a significant decrease in the number of VCMs was observed after acute CNO in mice that received the hM3Dq-mCherry vector, which was indicative of hM3Dq-mediated stimulation of iMSNs with CNO (Figure 8F, repeated measures 2-way ANOVA, drug: *F*_1,15_ = 10.14, *P* < 0.01, gene: *F*_1,15_ = 0.05, *P* = 0.82, interaction: *F*_1,15_ = 3.65, *P* = 0.08). In parallel, acute CNO increased the number of c-Fos^+^ neurons in the dorsal striatum in hM3Dq-expressing iMSNs (Figure 8G).

## Discussion

To our knowledge, this study shows for the first time that centrally acting antinociceptive and antipyretic acetaminophen mitigates orofacial dyskinesia induced by the long-term use of D2R antagonists. This finding was strongly supported by 2 independent clinical big data sources and by in vivo data from a conventional dyskinesia rodent model of haloperidol-induced VCMs. The antidyskinetic action was mediated via the stimulation of striatal TRPV1 with an acetaminophen metabolite, AM404, and was independent of the hypothermic action of acetaminophen.

Data mining using the FAERS identified many unexpected drug-drug interactions as confounding factors for adverse events (5, 6). The association between drugs and adverse events was strong enough owing to the large size of the data set, regardless of the diversity of the clinical conditions. Several D2R antagonists caused specific signals for dyskinesia in more than tens of thousands of cases, which enabled further high-sensitivity data mining of confounding factors. Potent mitigating effects on dyskinesia were found to be associated with acetaminophen, aspirin, proton pump inhibitors, thyroxine, and diuretics, none of which were previously shown to be effective in rodent models or human patients with dyskinesia (3). The only exception was fentanyl, which was predicted to be effective as an anticholinergic agent in a previous analysis of the FAERS (22). Interestingly, most anticholinergics were ineffective in our analysis and tended to increase the incidence of D2R antagonist–induced dyskinesia (Supplemental Table 2), which is consistent with previous clinical observations (23). Although further studies are needed to understand the molecular mechanism underlying the mitigating effects of other concomitant drugs, herein we focused on the effect of acetaminophen because it presented the highest *z* score with every D2R antagonist assessed.

Chronological analysis using JMDC data provided a precise incidence rate and timeline of D2R antagonist–induced dyskinesia. Only a small, but significant, increase in IRR for dyskinesia was observed with metoclopramide, one of the most frequently prescribed drugs in Japan, supporting a previous review showing the minimum risk of metoclopramide (24). This contrasted with the highest ROR signal for dyskinesia in the FAERS, for which case reports of metoclopramide increased greatly after a black box warning was applied by lawyers (25), resulting in an overestimation of the risk of metoclopramide. The IRR for dyskinesia was high in the haloperidol and aripiprazole cohorts; however, the yearly incidence was less than 1%. These values were much lower than the prevalence reported previously (2), probably due to the cautious, short-term prescription of low-dose D2R antagonists in these cohorts. Since the cumulative dose was low and the administration period was short in the haloperidol cohort, we chose the dopamine receptor partial agonist aripiprazole as a representative antipsychotic for the detailed analysis. The administration period of acetaminophen was also short, with the interquartile range remaining within 1 month, probably reflecting the temporary use of acetaminophen for pain and fever. Nevertheless, a retrospective comparison of matched cohorts demonstrated that such a short administration of acetaminophen was effective in halving the 3-year incidence of aripiprazole-induced dyskinesia without changing cumulative aripiprazole doses.

Acetaminophen is one of the most popular and widely used drugs for the treatment of pain and fever, but its mode of action is complex (12). In the brain and spinal cord, acetaminophen is converted to AM404 after deacetylation to *p*-aminophenol and conjugation with arachidonic acid by FAAH (18). Acetaminophen undergoes hepatic conjugation with glucuronide and sulfate to form inactive metabolites that are eliminated in the urine. The remaining acetaminophen metabolite is oxidized by cytochrome P450 enzymes to form *N*-acetyl-*p*-benzoquinone imine (NAPQI), which causes liver injury. In the present study, the dose of acetaminophen was low enough to avoid hepatic and renal insufficiency but adequate to affect the haloperidol outcome.

The metabolites AM404 and NAPQI contribute differently to the therapeutic effects of acetaminophen. AM404 exerts its antinociceptive effect by stimulating TRPV1 in the brain (14), while NAPQI and its subproducts are involved in TRPA1-mediated antinociception in the spinal cord (26). The potent TRPA1 agonist NAPQI is also involved in the hypothermic action of high-dose acetaminophen (15), whereas in endotoxin-induced fever models, the antipyretic effect of acetaminophen is mediated via cyclooxygenase inhibition and reduced prostaglandin E_2_ production in the brain (27). Our data using TRPV1-KO and TRPA1-KO mice showed that TRPV1 is solely involved in the antidyskinetic action of acetaminophen, AM404, and capsaicin, while TRPA1 mediates the hypothermic action of acetaminophen, probably through NAPQI.

In addition to stimulating TRPV1, AM404 increases endocannabinoids by inhibiting the cellular uptake of anandamide (16) to activate cannabinoid type 1 (CB_1_) receptors. In turn, anandamide attenuates haloperidol-induced VCMs via activation of CB_1_ receptors (28). Similar antihyperkinetic actions of CB_1_/TRPV1 coagonists have been reported in a rat model of Huntington’s disease (29). Since endocannabinoids may be endogenous agonists of TRPV1 channels, it is conceivable that the cooperation between CB_1_ receptors and TRPV1 is responsible for the antidyskinetic action of anandamide. However, several studies on the role of anandamide have used FAAH-deficient mice or FAAH blockers to increase the level of endocannabinoids by abolishing their degrading enzyme. These studies need to be interpreted with caution since FAAH is required to produce AM404 from acetaminophen in the brain, and its elimination causes deficiency of AM404 after acetaminophen treatment (14, 18). In addition, AM404 activates TRPV1 at concentrations much lower than those necessary to inhibit the uptake of anandamide (17). In this context, we infer that the contribution of CB_1_ receptors to the antidyskinetic action of AM404 and acetaminophen may be minimal.

TD is believed to be caused by a chronic blockade of dopamine D2Rs, which leads to the upregulation and hypersensitization of D2Rs expressed in iMSNs of the dorsal striatum, resulting in their hyperinhibition (1). Our observations suggest that TRPV1 has an essential role in the acetaminophen-mediated activation of iMSNs in the dorsal striatum and its antidyskinetic action, suggesting that TRPV1 contributed to the regulation of the motor function by activating dorsostriatal iMSNs. This hypothesis is supported by results of optogenetic stimulation of dorsostriatal iMSNs, which showed diminished haloperidol-induced VCMs (30). We have confirmed this by showing that chemogenetic stimulation of dorsostriatal iMSNs decreased haloperidol-induced VCMs with increasing c-Fos signaling in iMSNs. Hence, activation of dorsostriatal iMSNs via TRPV1 may be the underlying molecular mechanism of the antidyskinetic effect of acetaminophen.

A growing number of studies suggest TRPV1 involvement in the striatum synaptic properties, likely holding bidirectional modulating effects. In the striatum, TRPV1 is expressed in both presynaptic terminals and postsynaptic medium spiny neurons. Presynaptic TRPV1 channels facilitate the excitatory inputs in both the dorsal and ventral striatum by increasing the glutamatergic release probability (31). In contrast, the stimulation of postsynaptic TRPV1 in ventral striatal iMSNs is involved in the induction of long-term depression resulting from the endocytosis of AMPA receptors (32). Evidence suggests that the direction and magnitude of TRPV1-mediated regulation of excitatory inputs are influenced by TRPV1 levels and dopamine signaling, representing the cell type–specific and pathway-specific regulation of excitatory inputs via TRPV1, as shown in the ventral striatum (33). Because of the regional heterogeneity of glutamatergic inputs, gene expression, and dopaminergic signaling between the dorsal and ventral striatum (34), further studies on pathway-specific synaptic control via dorsostriatal TRPV1 are needed to clarify the synaptic mechanisms of TRPV1-mediated activation of dorsostriatal iMSNs.

In conclusion, our findings demonstrate that acetaminophen is effective in decreasing D2R antagonist–induced dyskinesia in both human retrospective analysis and experimental animal models. This combinatorial approach of drug repurposing based on clinical data will provide clues for new treatments with high clinical predictability and a well-defined molecular mechanism. Since long-term use of acetaminophen may cause NAPQI-induced liver toxicity and/or abuse or overdose of the cannabinoid mimetic acetaminophen, a novel, nonirritating compound selectively stimulating the central TRPV1 would be preferable.

## Methods

### Analysis of FAERS database.

Adverse event reports from 2004 to 2018 were obtained from the FDA website (35). Duplicated reports (among a total of 11,904,706 cases) were eliminated as previously reported (36), and the remaining 9,948,368 reports were analyzed. Arbitrary drug names, including trade names and abbreviations, were manually annotated to unified generic names using the Medical Subject Headings descriptor ID. Reports of dyskinesia were defined by the preferred terms “dyskinesia” and “tardive dyskinesia” in MedDRA (version 22.0). Analysis of FAERS data was performed as previously described (6). In volcano plots, *z* scores were used instead of *P* values to save space.

### Analysis of JMDC claims data.

Insurance claims data from January 2005 to March 2018 were purchased from JMDC Inc. The data set contained the monthly medical diagnosis and prescription claims of 5,550,241 employees and their dependents. Because of the features of the employee population and the national health insurance system in Japan, the patients were mostly aged 65 years and younger, and no patients aged 75 years or older were included in the analysis.

Individual diagnoses were assigned according to the International Classification of Diseases 10 (ICD-10). Cases of dyskinesia were defined by the ICD-10 standard disease name containing “dyskinesia,” including “tardive dyskinesia,” “orofacial dyskinesia,” and “oral dyskinesia,” but excluding dystonia, akathisia, tremor, parkinsonism, and other hyperkinetic symptoms. In the propensity score matching, the following categorization was used for risk factors: mood disorder = F30–F39, alcohol, substance abuse/dependence = F10–F19, diabetes mellitus = E10–E14, and hepatic disease = K70–K77. To identify patients who were prescribed D2R antagonists, we defined the aripiprazole cohort as those who received drugs belonging to the ATC code N05AX12. In the haloperidol cohort, patients who received haloperidol injections only (*n* = 9892) were excluded, and those who routinely received haloperidol or its decanoate ester were included (*n* = 6053). Similarly, patients who only occasionally took metoclopramide were excluded (*n* = 398,512), whereas those who routinely received metoclopramide were included (*n* = 302,064). The acetaminophen cohort comprised patients who received drugs belonging to any of the following ATC codes: N02BE01, R05X, N02AA58, N02BE71, or N02AJ13. The following categorization was used for propensity score matching: antiparkinsonian drugs (N04) and additional antipsychotic drugs (N05A).

Analyses of the JMDC data were performed using the R v4.0.2 and R studio v1.3.959 software (R Foundation for Statistical Computing). The R packages survival and MatchIt were used to perform time-series analyses. The incidence of dyskinesia according to D2R antagonist use was first evaluated using the Poisson regression, and the results were expressed as the IRR along with the 95% CI and *z* scores. After each D2R antagonist cohort was divided into 2 groups (with and without acetaminophen), 1:1 propensity score matching (37) was used to eliminate the deflections in the number of patients who had risk factors. The propensity score–matched pairs were created by matching 2 groups using the nearest-neighbor method with a 0.2 (haloperidol) or 0.01 (aripiprazole and metoclopramide) caliper width (38). The resulting cohorts were analyzed for causality by sequence symmetry analysis during an observation period of 36 months, as described previously (39). Using the matched cohort pairs, the daily and cumulative doses, and administration periods of D2R antagonists and acetaminophen, were quantified and compared. Cumulative incidences of dyskinesia were compared between the cohorts with and without acetaminophen by conventional survival analysis (40), and the survival curves were represented by Kaplan-Meier plots. Statistical significance was evaluated using the log-rank test and Cox proportional regression analysis to calculate hazard ratios. The number at risk indicates the number of patients who may have an onset of dyskinesia each month.

### Animals.

All experiments were designed to minimize the use of animals and the number of experiments. Male Wistar rats (9 weeks old, 200–250 g) and male C57BL/6J mice (6–7 weeks old, 20–30 g) were purchased from Japan SLC. TRPV1-KO mice (B6.129X1-Trpv1^tm1Jul^/J, RRID: IMSR_JAX:03770) were originally provided by Julius (41). TRPA1-KO mice (B6.129P-Trpa1^tm1Kykw^/J, RRID: IMSR_JAX:006401) were purchased from The Jackson Laboratory. *Adora2A*-Cre mice [B6.FVB(Cg)-Tg(Adora2a-cre)KG139sat/Mmucd, RRID: MMRRC_036158-UCD] were purchased from the Mutant Mouse Regional Resource Center. These genetically modified mice on a C57BL/6J background were maintained in our laboratory. All animals were housed at a constant ambient temperature (22°C ± 2°C) on a 12-hour light/12-hour dark cycle, with free access to food and water.

### Drugs and reagents.

Haloperidol was purchased from Tokyo Chemical Industry; acetaminophen and capsaicin from Nacalai Tesque; AM404 from Alomone Labs; methamphetamine from Sumitomo Dainippon Pharma; CNO from Cayman Chemical; DL-2-amino-5-phosphonopentanoic acid (DL-APV) from MilliporeSigma; dinitroquinoxaline-2,3(*1H,4H*)-dione (DNQX) from Tocris Bioscience (Bio-Techne); bicuculline from Enzo Life Science; and pentobarbital from Kyoritsu Seiyaku.

Haloperidol (1 mg/kg for rats, 2 mg/kg for mice) and acetaminophen (50 or 100 mg/kg for rats, 300 mg/kg for mice) were suspended in 0.5% carboxymethyl cellulose. AM404 was dissolved in 20% dimethyl sulfoxide (DMSO) and diluted with saline. Capsaicin was dissolved in DMSO with 1% Tween 80 and diluted in saline (for rats) or 5% DMSO with 1% Tween 80 and diluted in saline (for mice). Methamphetamine was dissolved in saline prior to use. CNO was dissolved in 1% DMSO and diluted in saline for behavioral analysis or in artificial cerebrospinal fluid (ACSF) for electrophysiological analysis.

### Rat intracerebroventricular injection.

Rats were anesthetized with 60 mg/kg pentobarbital and placed in a stereotaxic apparatus, with bregma and lambda kept in the same horizontal plane. A hole was drilled into their skulls, and a stainless steel guide cannula (Eicom) was inserted into the right lateral ventricle. Based on a rat brain atlas, the following stereotaxic coordinates were used: anterior-posterior (AP) = +0.6 mm, medial-lateral (ML) = +1.6 mm, dorsal-ventral (DV) = +4.5 mm from bregma. The guide cannula was implanted and fixed with dental acrylic, and a 30-gauge stainless steel stylet was placed into the guide cannula to prevent the entry of foreign materials. The experimental procedure was started 3–5 days after the surgery.

An injector (30 gauge) was fitted into the guide cannula, and intracerebroventricular infusions were made using 10 μL microsyringes (Hamilton Company) attached to the injector with a polyethylene tube. AM404 (10 or 50 nmol) or the control vehicle was injected with an automatic infusion pump at a rate of 1 μL/min and a total injection volume of 5 μL. After the experiments, Evans blue solution (5 μL) was injected through the cannula to confirm the injection site. If the injection site was incorrect, the animal was excluded from the analysis.

### Mouse microinjection.

Mice were anesthetized with sodium pentobarbital (50 mg/kg) injected intraperitoneally and implanted with a bilateral guide cannula directed at the dorsal striatum (AP = +0.3 mm, ML = ±2.6 mm, DV = +3.0 mm from bregma, angled 10°) and fixed to the skull by dental cement. On the day of the experiment, the injection cannula was inserted into the guide, and the drug (0.5 or 1 pmol AM404) was injected at a rate of 0.25 μL/min in a total injection volume of 1 μL. After injection, the injection cannula was left in place during the recording. After the experiments, Evans blue solution (0.5 μL) was injected through the cannula to confirm the injection site. If the injection site was incorrect, the animal was excluded from the analysis.

### Preparation and delivery of AAV vectors.

pAAV-hSyn-DIO-hM3D(Gq)-mCherry was obtained from Addgene (plasmid 44361). The AAV vector was prepared as described previously (42). Briefly, Lenti-X 293T cells were transfected with pAAV-hSyn-DIO-hM3D, pAAV-DJ, and pHelper using polyethylenimine (Max, Polysciences). After 72 hours of transfection, the cells were gently collected and freeze-thawed 4 times to break the cell membrane. DNA and RNA were removed using benzonase nuclease (MilliporeSigma). After digestion, the solution was centrifuged at 17,781*g* for 10 minutes, and the supernatant was collected. This procedure was repeated at least 5 times to remove cell debris. *Adora2A*-Cre mice (6–7 weeks old, 20–30 g) were anesthetized with sodium pentobarbital and fixed on a small animal stereotaxic frame (Narishige). AAV-hSyn-DIO-hM3D(Gq)-mCherry or AAV-hSyn-DIO-mCherry (0.25 μL/side) was bilaterally microinjected into the dorsal striatum (AP = +0.6 mm, ML = ±2.4 mm, DV = +3.2 mm from bregma). At least 4 weeks after the viral injection, the mice were used for behavioral tests or electrophysiological recordings.

### Haloperidol-induced VCMs.

In the rat model (9), haloperidol, acetaminophen, or both were orally administered once daily for 21 days. Approximately 24 hours after the last treatment, the rats were individually placed in clear-walled cages (10 × 20 × 30 cm), and the number of VCMs in a 3-minute period was counted. Habituation was performed for 30 minutes on the previous 3 days and another 30 minutes immediately before the evaluation. During the observation session, 2 video cameras were placed on both sides of the cage to record the behavior of the rats. The VCMs were defined as single mouth openings in the vertical plane not directed toward a physical material and counted in a blinded manner.

In the mouse model, after 21 days of oral treatment with haloperidol, the mice were orally administered acetaminophen or the vehicle and individually placed in a transparent head fixation chamber (28 mm diameter). Habituation to the chamber was performed for 30 min/d for 3 days before the evaluation. Beginning 60 minutes after drug administration, the behavior of the mice was recorded using a video camera placed underneath the mice. The number of VCMs was counted over a 3-minute period 3 times in a blinded manner, and the average number of VCMs was analyzed.

### Repetitive VCM counting with microinjection into the dorsal striatum.

WT and TRPV1-KO mice were orally administered haloperidol (2 mg/kg/d) for 21 days. On day 22, after the basal VCMs were counted once for 5 minutes, half of the mice randomly were assigned to receive the vehicle (group 1), or the test drug (group 2), through a preimplanted cannula in the dorsal striatum. Five minutes later, VCMs were counted again for 5 minutes to determine the effect of the injected drug. Daily haloperidol treatment was continued for 4 more days, and on day 26, the second VCM counting was performed in a crossover design with the test drug injection to group 1 and vehicle injection to group 2. After the experiment, the application sites were confirmed using Evans blue staining.

### Immunohistochemistry.

Mice were treated with haloperidol (2 mg/kg/d) or vehicle orally administrated for 21 days and singly housed for 3 days before perfusion. On the test day, after treatment with acetaminophen or vehicle, the mice were anesthetized with pentobarbital and transcardially perfused with phosphate-buffered saline followed by 4% paraformaldehyde (Nacalai Tesque) in phosphate buffer. After fixation, the brains were stored in the fixative for 90 minutes, transferred to 15% sucrose in 0.1 M phosphate buffer for 24 hours, and frozen using dry ice. The brains were cryosectioned into 30 μm thick coronal sections with a cryostat (Leica CM3050S; Leica Biosystems) and stored at −80°C.

For c-Fos immunohistochemistry, the sections were immersed in phosphate-buffered saline containing 0.25% Triton X-100 (Nacalai Tesque) for permeabilization and incubated overnight at room temperature with mouse monoclonal anti–c-Fos antibody (1:500; NBP2-50037, Novus Biologicals) and rabbit monoclonal anti-ppENK antibody (1:500; RA14124, Neuromics), followed by incubation with Alexa Fluor 594–labeled donkey anti-mouse IgG (1:200; A21203, Invitrogen, Thermo Fisher Scientific) and Alexa Fluor 488–labeled donkey anti-rabbit IgG (1:200; A21206, Invitrogen, Thermo Fisher Scientific) for 1.5 hours at room temperature in the dark. Additionally, the sections were immersed in 0.25% Triton X-100 for permeabilization and then incubated overnight at room temperature with mouse monoclonal anti–c-Fos antibody, followed by incubation with Alexa Fluor 488–labeled donkey anti-mouse IgG (1:200; A21202, Invitrogen, Thermo Fisher Scientific) for 1.5 hours at room temperature in the dark. Images were captured using a confocal fluorescence microscope (Fluoview FV10i; Olympus). The number of c-Fos^+^ppENK^+^ cells in a 0.045 mm^2^ field of the dorsal striatum, 0.3 mm anterior to the bregma, was counted. Counting was performed in the 4 regions.

### Chemogenetic activation of iMSNs and haloperidol-induced VCMs.

Intact *Adora2A*-Cre mice received a viral vector carrying hM3Dq-mCherry or mCherry alone through a cannula in the dorsal striatum on day 1 and orally administrated haloperidol (2 mg/kg/d) for 21 days from day 8. On day 29, half of the mice received vehicle (group 1) intraperitoneally, and the rest of the mice received 0.5 mg/kg CNO (group 2). After 30 minutes, the number of VCMs was counted 3 times for 3 minutes. The daily haloperidol treatment was continued for 4 more days, and on day 33, the second VCM measurement was performed in a crossover design, with CNO given to group 1 and the vehicle to group 2. After 4 days of daily haloperidol administration, c-Fos staining was performed.

### Recording neural activity.

Electrophysiological recordings were performed as previously described (43). Briefly, 4 weeks after AAV injection into *Adora2A*-Cre mice, coronal brain slices (200 μm thick) containing the striatum were prepared and allowed to recover in oxygenated ACSF (composition in mM: 124 NaCl, 3 KCl, 26 NaHCO_3_, 1 NaH_2_PO_4_, 2.4 mM CaCl_2_, 1.2 mM MgCl_2_, and 10 d-glucose, pH 7.3) at 32°C for at least 1 hour before recording. For recording, the chamber was kept at 27°C ± 1°C by continuous perfusion of oxygenated ACSF containing DNQX (20 μM), DL-APV (50 μM), and bicuculline (20 μM). The resistance of the electrodes was 3–7 MΩ when filled with the internal solution (composition in mM: 140 K-gluconate, 5 KCl, 10 HEPES, 2 Na-ATP, 2 MgCl_2_, and 0.2 EGTA, pH 7.3, adjusted with KOH). The series resistance was compensated by 70% and was maintained within 35 MΩ. The firing activity of hM3Dq-expressing neurons in the dorsal striatum was recorded using the current injection method before and after CNO application (3 μM, 5 minutes).

### Statistics.

Statistical analyses of the animal experiments using 1- or 2-way ANOVA and 2-tailed *t* test were performed using Prism v9.1.0 (GraphPad Software). For post hoc 1-way and 2-way ANOVA tests, Tukey’s and Sidak’s, respectively, multiple comparisons tests were used. *P* < 0.05 was considered statistically significant. These data are presented as mean ± SEM.

### Study approval.

All retrospective observational studies were approved by the Ethics Committee of Kyoto University Faculty of Medicine (number R1018-1). All animal experiments were approved by the Kyoto University Animal Research Committee in accordance with the ethical guidelines (numbers 14-42-4, 20-42).

## Author contributions

TN and SK designed the project. TN, CT, GK, and HY performed the clinical data analysis. K Nagaoka and SS performed the animal experiments. NA performed electrophysiological recordings. K Nagayasu, YF, K Kuroda, K Kaibuchi, YM, and HS provided materials and technical advice. K Nagaoka, HY, NA, and SK analyzed the data and wrote the manuscript.

## Supplementary Material

Supplemental data

Supplemental Table 1

Supplemental Table 2

## Version 1 04/15/2021

In-Press Preview

## Version 2 05/24/2021

Electronic publication

## References

[1] Stahl SM (2017). Neuronal traffic signals in tardive dyskinesia: not enough “stop” in the motor striatum. CNS Spectr.

[2] Carbon M (2017). Tardive dyskinesia prevalence in the period of second-generation antipsychotic use: a meta-analysis. J Clin Psychiatry.

[3] Salem H (2017). Antipsychotic-induced tardive dyskinesia: from biological basis to clinical management. Expert Rev Neurother.

[4] Khorassani F (2020). Valbenazine and deutetrabenazine: vesicular monoamine transporter 2 inhibitors for tardive dyskinesia. Am J Health Syst Pharm.

[5] Zhao S (2013). Systems pharmacology of adverse event mitigation by drug combinations. Sci Transl Med.

[6] Nagashima T (2016). Prevention of antipsychotic-induced hyperglycaemia by vitamin D: a data mining prediction followed by experimental exploration of the molecular mechanism. Sci Rep.

[7] Michel C (2017). Can disproportionality analysis of post-marketing case reports be used for comparison of drug safety profiles?. Clin Drug Investig.

[8] Turrone P (2002). The vacuous chewing movement (VCM) model of tardive dyskinesia revisited: is there a relationship to dopamine D_2_ receptor occupancy?. Neurosci Biobehav Rev.

[9] Naidu PS (2002). Carvedilol attenuates neuroleptic-induced orofacial dyskinesia: possible antioxidant mechanism. Br J Pharmacol.

[10] Jaeschke H (2014). Acetaminophen-induced liver injury: from animal models to humans. J Clin Transl Hepatol.

[11] Mazer M, Perrone J (2008). Acetaminophen-induced nephrotoxicity: pathophysiology, clinical manifestations, and management. J Med Toxicol.

[12] Bertolini A (2006). Paracetamol: new vistas of an old drug. CNS Drug Rev.

[13] Mezey E (2000). Distribution of mRNA for vanilla receptor subtype 1 (VR1), and VR1-like immunoreactivity, in the central nervous system of the rat and human. Proc Natl Acad Sci U S A.

[14] Mallet C (2010). TRPV1 in brain is involved in acetaminophen-induced antinociception. PLoS One.

[15] Gentry C (2015). TRPA1 mediates the hypothermic action of acetaminophen. Sci Rep.

[16] Beltramo M (1997). Functional role of high-affinity anandamide transport, as revealed by selective inhibition. Science.

[17] De Petrocellis L (2000). Overlap between the ligand recognition properties of the anandamide transporter and the VR1 vanilloid receptor: inhibitors of anandamide uptake with negligible capsaicin-like activity. FEBS Lett.

[18] Hogestatt ED (2005). Conversion of acetaminophen to the bioactive N-acylphenolamine AM404 via fatty acid amide hydrolase-dependent arachidonic acid conjugation in the nervous system. J Biol Chem.

[19] Calabresi P (2014). Direct and indirect pathways of basal ganglia: a critical reappraisal. Nat Neurosci.

[20] Armbruster BN (2007). Evolving the lock to fit the key to create a family of G protein-coupled receptors potently activated by an inert ligand. Proc Natl Acad Sci U S A.

[21] Belleau ML, Warren RA (2000). Postnatal development of electrophysiological properties of nucleus accumbens neurons. J Neurophysiol.

[22] Xu D (2017). MSBIS: a multi-step biomedical informatics screening approach for identifying medications that mitigate the risks of metoclopramide-induced Tardive dyskinesia. EBioMedicine.

[23] Desmarais JE (2012). Anticholinergics in the era of atypical antipsychotics: short-term or long-term treatment?. J Psychopharmacol.

[24] Al-Saffar A (2019). Gastroparesis, metoclopramide, and tardive dyskinesia: risk revisited. Neurogastroenterol Motil.

[25] Ehrenpreis ED (2013). The metoclopramide black box warning for tardive dyskinesia: effect on clinical practice, adverse event reporting, and prescription drug lawsuits. Am J Gastroenterol.

[26] Andersson DA (2011). TRPA1 mediates spinal antinociception induced by acetaminophen and the cannabinoid ^Δ^9-tetrahydrocannaniorcol. Nat Commun.

[27] Ayoub SS, Flower RJ (2019). Loss of hypothermic and anti-pyretic action of paracetamol in cyclooxygenase-1 knockout mice is indicative of inhibition of cyclooxygenase-1 variant enzymes. Eur J Pharmacol.

[28] Röpke J (2014). Anandamide attenuates haloperidol-induced vacuous chewing movements in rats. Prog Neuropsychopharmacol Biol Psychiatry.

[29] de Lago E (2005). Arvanil, a hybrid endocannabinoid and vanilloid compound, behaves as an antihyperkinetic agent in a rat model of Huntington’s disease. Brain Res.

[30] Bordia T (2016). Striatal cholinergic interneurons and D2 receptor-expressing GABAergic medium spiny neurons regulate tardive dyskinesia. Exp Neurol.

[31] Musella A (2009). TRPV1 channels facilitate glutamate transmission in the striatum. Mol Cell Neurosci.

[32] Grueter BA (2010). Postsynaptic TRPV1 triggers cell-type specific LTD in the nucleus accumbens. Nat Neurosci.

[33] Deroche MA (2020). Cell-type- and endocannabinoid-specific synapse connectivity in the adult nucleus accumbens core. J Neurosci.

[34] Puighermanal E (2020). Functional and molecular heterogeneity of D2R neurons along dorsal ventral axis in the striatum. Nat Commun.

[35] US FDA. Questions and Answers on FDA’s Adverse Event Reporting System (FAERS). https://www.fda.gov/drugs/surveillance/questions-and-answers-fdas-adverse-event-reporting-system-faers Accessed April 23, 2021

[36] Banda JM (2016). A curated and standardized adverse drug event resource to accelerate drug safety research. Sci Data.

[37] Olmos A, Govindasamy P (2015). Propensity scores: a practical introduction using R. J Multidiscrip Eval.

[38] Austin PC (2011). Optimal caliper widths for propensity-score matching when estimating differences in means and differences in proportions in observational studies. Pharm Stat.

[39] Lai EC (2017). Sequence symmetry analysis in pharmacovigilance and pharmacoepidemiologic studies. Eur J Epidemiol.

[40] Yokoyama S (2018). Bleeding risk of warfarin and direct oral anticoagulants in younger population: a historical cohort study using a Japanese claims database. Int J Med Sci.

[41] Caterina MJ (2000). Impaired nociception and pain sensation in mice lacking the capsaicin receptor. Science.

[42] Deguchi Y (2016). mDia and ROCK mediate actin-dependent presynaptic remodeling regulating synaptic efficacy and anxiety. Cell Rep.

[43] Asaoka N (2019). An adenosine A_2A_ receptor antagonist improves multiple symptoms of repeated quinpirole-induced psychosis. eNeuro.

