# Supplementary material for: Striatal TRPV1 activation by acetaminophen ameliorates dopamine D2 receptor antagonist–induced orofacial dyskinesia
Source: JCI Insight. 2021 May 24;6(10):e145632. doi: 10.1172/jci.insight.145632 (PMC8262333; doi:10.1172/jci.insight.145632)
Supplement: Supplemental data [file jciinsight-6-145632-s039.pdf]

Supplemental Material

Striatal TRPV1 activation by acetaminophen ameliorates dopamine D_2 receptor antagonists-induced orofacial dyskinesia

Koki Nagaoka[1], Takuya Nagashima[1], Nozomi Asaoka[1,2], Hiroki Yamamoto[1], Chihiro Toda[1], Gen Kayanuma[1], Soni Siswanto[1], Yasuhiro Funahashi[3,4], Keisuke Kuroda[3], Kozo Kaibuchi[3,4], Yasuo Mori[5], Kazuki Nagayasu[1], Hisashi Shirakawa[1], and Shuji Kaneko[1]

[1] Department of Molecular Pharmacology, Graduate School of Pharmaceutical Sciences, Kyoto University, Kyoto, Japan

[2] Department of Pharmacology, Kyoto Prefectural University of Medicine, Kyoto, Japan

[3] Department of Cell Pharmacology, Graduate School of Medicine, Nagoya University, Nagoya, Japan

[4] Research Project for Neural and Tumor Signaling, Institute for Comprehensive Medical Science, Fujita Health University, Toyoake, Japan.

[5] Department of Synthetic Chemistry and Biological Chemistry, Graduate School of Engineering, Kyoto University, Katsura Campus, Kyoto, Japan.

Address correspondence to: Shuji Kaneko (skaneko@pharm.kyoto-u.ac.jp)

Supplemental Table 1. Overall results of disproportionality analysis for dyskinesia in the FDA Adverse Event Reporting System (FAERS) data

Filename: 145632-INS-RG-TR-2_sd_471796.xlsx

Individuals in the FAERS data were divided into the following four groups: (a) individuals who received the drug of interest (drug A) and exhibited dyskinesia; (b) individuals who received the drug A but did not exhibit dyskinesia; (c) individuals who did not receive the drug A and exhibited dyskinesia; and (d) individuals who did not receive the drug A and did not exhibit dyskinesia. The reporting odds ratio (ROR) with a 95% confidence interval (CI) and *Z* score were calculated as per the following equations:

$$\mathrm{ROR} = \frac{a/b}{c/d} \qquad (1)$$

$$95\%\ \mathrm{CI} = \exp\left\{\log(\mathrm{ROR}) \pm 1.96\sqrt{\frac{1}{a}+\frac{1}{b}+\frac{1}{c}+\frac{1}{d}}\right\} \qquad (2)$$

$$\mathrm{Z\ score} = \frac{\log(\mathrm{ROR})}{\sqrt{\frac{1}{a}+\frac{1}{b}+\frac{1}{c}+\frac{1}{d}}} \qquad (3)$$

where *a*, *b*, *c*, and *d* refer to the number of individuals in each group.

Supplemental Table 2. Overall confounding effects of the concomitant drug (drug B) on dyskinesia associated with haloperidol, aripiprazole, or metoclopramide in the FDA Adverse Event Reporting System (FAERS) data

Filename: 145632-INS-RG-TR-2_sd_471797.xlsx (Three separate tab sheets)

Individuals who received haloperidol, aripiprazole, or metoclopramide (drug A) were divided into the following four groups: (a1) individuals who received the concomitant drug of interest (drug B) and exhibited dyskinesia; (b1) individuals who received the drug B but did not exhibit dyskinesia; (c1) individuals who did not receive the drug B and exhibited dyskinesia; and (d1) individuals who did not receive the drug B and did not exhibit dyskinesia. The reporting odds ratio (ROR) with a 95% confidence interval (CI) and *Z* score for the drug A-induced dyskinesia was calculated as per the following equations:

$$\mathrm{ROR} = \frac{a1/b1}{c1/d1} \qquad (4)$$

$$95\%\ \mathrm{CI} = \exp\left\{\log(\mathrm{ROR}) \pm 1.96\sqrt{\frac{1}{a1}+\frac{1}{b1}+\frac{1}{c1}+\frac{1}{d1}}\right\} \qquad (5)$$

$$\mathrm{Z\ score} = \frac{\log(\mathrm{ROR})}{\sqrt{\frac{1}{a1}+\frac{1}{b1}+\frac{1}{c1}+\frac{1}{d1}}} \qquad (6)$$

where *a1*, *b1*, *c1*, and *d1* refer to the number of individuals in each group.

Supplemental Table 3. Incidence rate ratio (IRR) of drug-induced dyskinesia in the JMDC claims data

Haloperidol	Cases	Incidence (% per person-year)	IRR (95% CI)	*Z* score	$-\log_{10}P$
+	11/3,104	0.182	70.1 (38.4-127)	13.9	42.9
−	320/5,211,148	0.003			

Aripiprazole	Cases	Incidence (% per person-year)	IRR (95% CI)	*Z* score	$-\log_{10}P$
+	70/29,512	0.134	61.0 (46.9-79.4)	30.6	205
−	268/5,177,545	0.002			

Metoclopramide	Cases	Incidence (% per person-year)	IRR (95% CI)	*Z* score	$-\log_{10}P$
+	38/269,889	0.007	2.51 (1.79-3.51)	5.35	7.06
−	313/4,916,587	0.003			

Supplemental Table 4. Propensity score matching of the cohorts taking D_2 receptor antagonists in the JMDC claims data

Population with haloperidol	Before matching			After matching		
	Without acetaminophen	With acetaminophen	*P* value	Without acetaminophen	With acetaminophen	*P* value
Total	1,274	1,830	–	1,265	1,265	–
Elderly (≥ 65 years)	73	93	0.48	68	70	0.93
Female	610	949	0.03	605	601	0.90
Antiparkinsonian drug	653	1,076	3.72×10^{-5}	652	662	0.72
Additional antipsychotic drug	798	1,337	9.07×10^{-10}	797	833	0.15
Mood disorder	682	1,122	1.83×10^{-5}	676	683	0.81
Alcohol, substance abuse/dependence	70	112	0.51	68	77	0.49
Diabetes mellitus	477	875	1.22×10^{-8}	476	483	0.81
Hepatic disease	405	844	1.56×10^{-15}	405	410	0.86

Population with aripiprazole	Before matching			After matching		
	Without acetaminophen	With acetaminophen	*P* value	Without acetaminophen	With acetaminophen	*P* value
Total	13,781	15,731	–	12,216	12,216	–
Elderly (≥ 65 years)	225	189	1.98×10^{-3}	149	147	0.95
Female	6,245	7,465	2.50×10^{-4}	5,538	5,457	0.30
Antiparkinsonian drug	1,979	2,899	$< 2.20 \times 10^{-16}$	1,889	1,823	0.25
Additional antipsychotic drug	5,352	7,691	$< 2.20 \times 10^{-16}$	5,219	5,178	0.60
Mood disorder	10,997	12,704	0.04	9,628	9,722	0.14
Alcohol, substance abuse/dependence	362	644	5.31×10^{-12}	332	352	0.46
Diabetes mellitus	4,013	6,303	$< 2.20 \times 10^{-16}$	3,947	3,955	0.92
Hepatic disease	3,872	6,578	$< 2.20 \times 10^{-16}$	3,850	3,889	0.60

Population with metoclopramide	Before matching			After matching		
	Without acetaminophen	With acetaminophen	*P* value	Without acetaminophen	With acetaminophen	*P* value
Total	65,479	204,410	–	65,464	65,464	–
Elderly (≥ 65 years)	3,002	4,917	$< 2.20 \times 10^{-16}$	2,989	2,989	1.00
Female	35,832	106,451	$< 2.20 \times 10^{-16}$	35,823	35,835	0.95
Antiparkinsonian drug	542	2,621	$< 2.20 \times 10^{-16}$	538	496	0.20
Antipsychotic drug	2,168	8,883	$< 2.20 \times 10^{-16}$	2,161	2,126	0.60
Mood disorder	5,204	19,591	$< 2.20 \times 10^{-16}$	5,202	5,184	0.86
Alcohol, substance abuse/dependence	714	2,546	1.68×10^{-3}	704	711	0.87
Diabetes mellitus	11,290	41,865	$< 2.20 \times 10^{-16}$	11,289	11,321	0.82
Hepatic disease	12,269	51,412	$< 2.20 \times 10^{-16}$	12,264	12,307	0.77

The number of patients in each group is shown.

Supplemental Table 5. Daily and cumulative doses, and administration periods of D_2 receptor antagonists and acetaminophen in the propensity score-matched cohorts selected from the JMDC claims data

Matched haloperidol cohort	Without acetaminophen		With acetaminophen			
	Haloperidol		Haloperidol		Acetaminophen	
	Median (IQR)	Range	Median (IQR)	Range	Median (IQR)	Range
Mean daily dose (mg)	1.5 (0.8-3)	0.1-54	1.5 (0.8-3)	0.1-56	600 (450-1,045)	0.8-9,750
Cumulative dose (mg)	80 (21-392)	0.4-33,471	91 (25-482)	0.6-34,784	6,250 (2,650-15,350)	4-1,678,725
Administration period (day)	52 (14-198)	1-4,267	63 (18-284)	1-7,407	10 (5-24)	1-1,456

Matched aripiprazole cohort	Without acetaminophen		With acetaminophen			
	Aripiprazole		Aripiprazole		Acetaminophen	
	Median (IQR)	Range	Median (IQR)	Range	Median (IQR)	Range
Mean daily dose (mg)	3 (2.5-6)	0.1-360	3 (2.3-5.7)	0.1-321	675 (450-1,200)	0.8-36,000
Cumulative dose (mg)	306 (84-1,113)	1.8-84,870	414 (90-1,647)	0.7-104,124	5,175 (2,400-11,300)	4-2,970,325
Administration period (day)	98 (28-293)	1-5,716	142 (30-465)	1-6,889	7 (4-16)	1-2,306

Matched metoclopramide cohort	Without acetaminophen		With acetaminophen			
	Metoclopramide		Metoclopramide		Acetaminophen	
	Median (IQR)	Range	Median (IQR)	Range	Median (IQR)	Range
Mean daily dose (mg)	15 (15-15)	0.3-60	15 (12-15)	0.2-100	643 (450-1,083)	20-39,900
Cumulative dose (mg)	60 (45-105)	0.9-48,300	69 (45-120)	0.2-79,020	6,100 (2,960-13,300)	60-3,366,625
Administration period (day)	4 (3-7)	1-2,055	5 (3-8)	1-4,959	10 (4-21)	1-2,955

The median value, interquartile range (IQR), and minimum-maximum ranges are shown for each group.

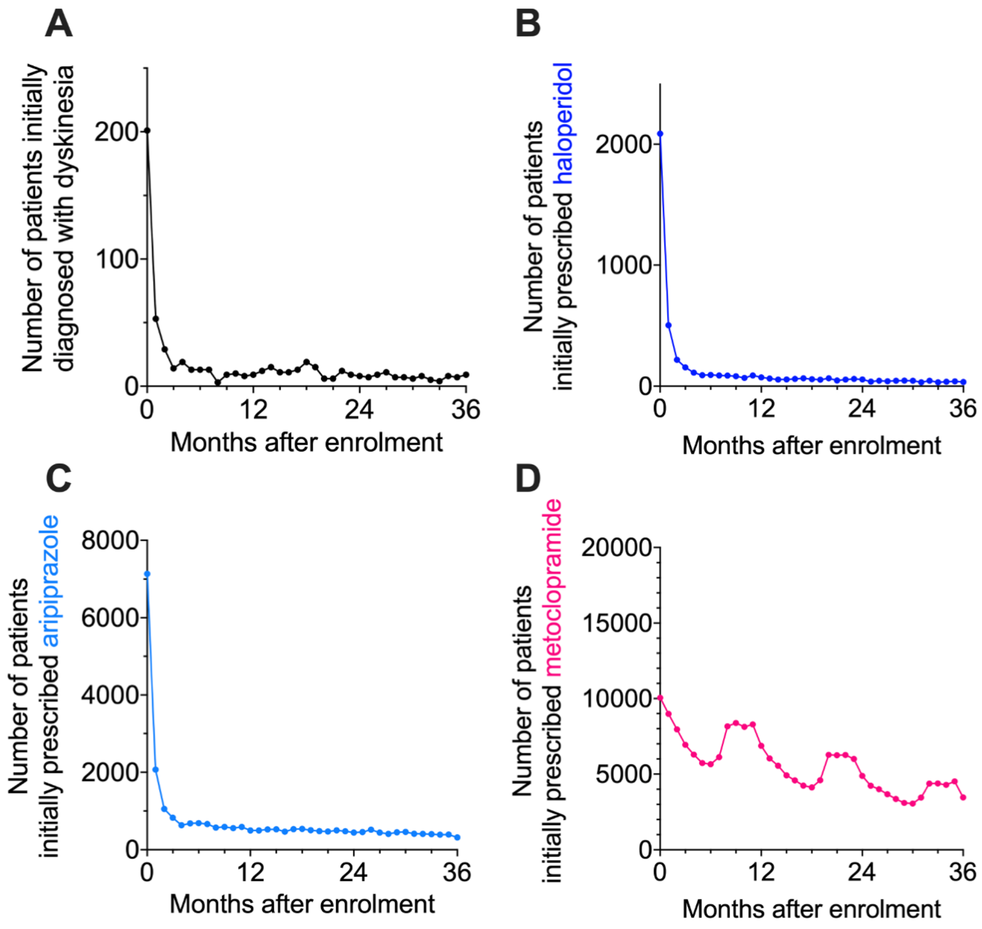

Supplemental Figure 1. Time distribution of the first event after enrolment in the JMDC claims data. Time intervals from the insurance enrolment of a patient to the initial diagnosis of dyskinesia (**A**) and the first prescription of haloperidol (**B**), aripiprazole (**C**), or metoclopramide (**D**). The number of patients is shown on a monthly basis.

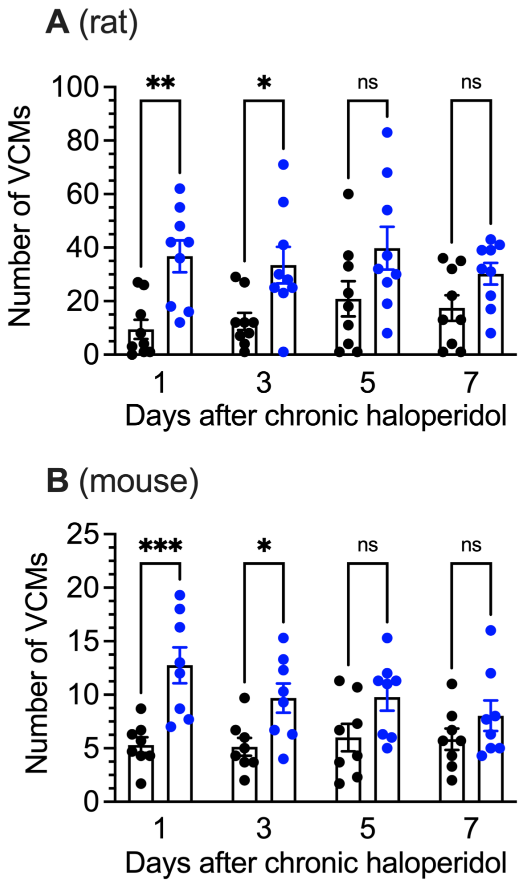

Supplemental Figure 2. Orofacial dyskinesia during the withdrawal phase of haloperidol after 21-day oral treatment in rodents. Rats (**A**, $n = 9$ per group) and mice (**B**, $n = 8$ per group) were treated with daily haloperidol administrated orally (1 and 2 mg/kg/day for rats and mice, respectively) for 21 days. The number of vacuous chewing movements (VCMs) was counted for 3 min from 24 h after the last treatment (day 1) at 2-days intervals. Individual data are shown as the mean ± SEM. Statistical significance was tested by two-way ANOVA (**A**, Time: $F_{3,48} = 0.86$, $P = 0.46$, Drug: $F_{1,16} = 20.6$, $P < 0.001$, Subject: $F_{16,48} = 1.34$, $P = 0.21$. **B**, Time: $F_{3,42} = 1.32$, $P = 0.28$, Drug: $F_{1,14} = 16.3$, $P < 0.01$, Subject: $F_{14,42} = 2.04$, $P = 0.038$) with post-hoc multiple comparisons. $^{*}P < 0.05$; $^{**}P < 0.01$; $^{***}P < 0.001$; ns, not significant.

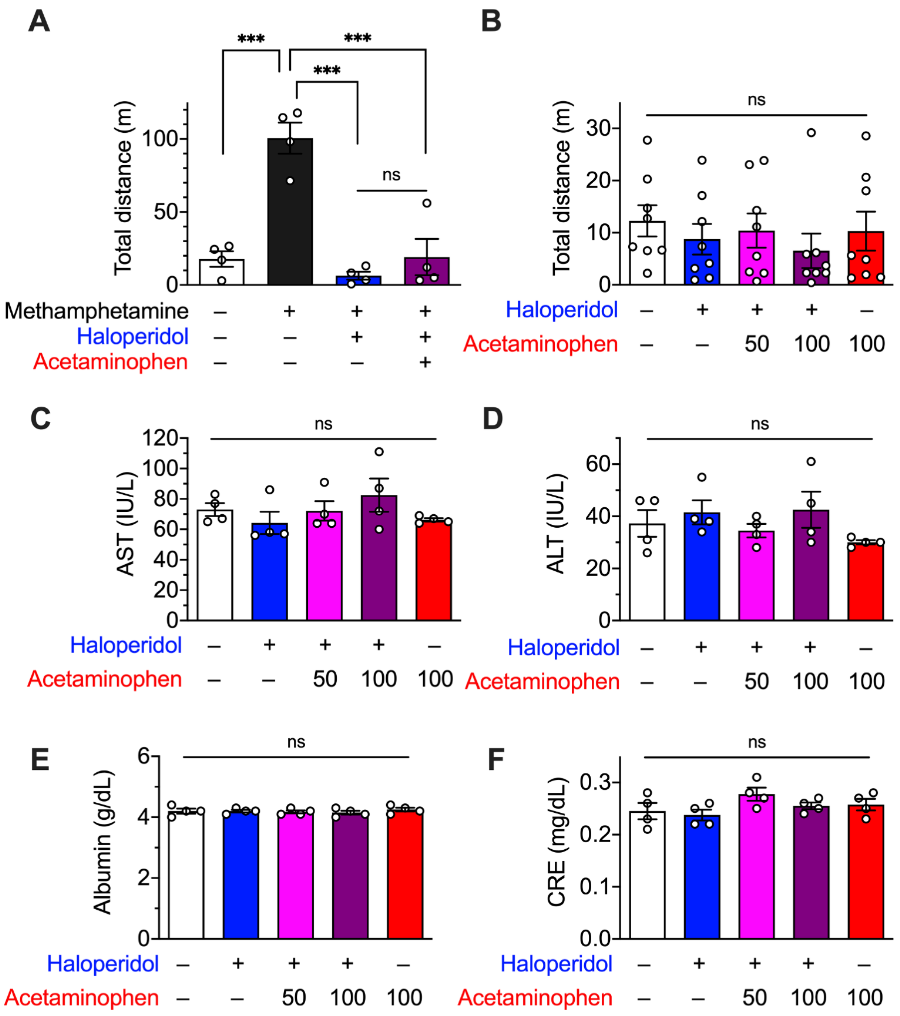

Supplemental Figure 3. Effects of acetaminophen on locomotor activity, antipsychotic action of haloperidol, and hepatic and renal function in rats. (**A**) Rats (n = 4 per group) were acutely treated with haloperidol (1 mg/kg), acetaminophen (100 mg/kg), or both, administrated orally; 45 min after which, methamphetamine (2 mg/kg) was injected intra-peritoneally. The locomotor activity of the rats was measured for 30 min, beginning 15 min after methamphetamine administration. (**B**) Rats (n = 8 per group) were treated daily with haloperidol (1 mg/kg/day), acetaminophen (50 or 100 mg/kg/day.), or both, administrated orally for 21 days. The locomotor activity of the rats was measured for 30 min beginning 24 h after the last treatment administration. (**C–F**) Rats (n = 4 per group) were treated daily with haloperidol (1 mg/kg/day), acetaminophen (50 or 100 mg/kg/day), or both, administrated orally for 21 days, and blood was sampled from the heart 24 h after the last treatment. Serum levels of aspartate aminotransferase (AST, **C**), alanine aminotransferase (ALT, **D**), albumin (**E**), and creatinine (CRE, **F**) were measured. Individual data are shown as the mean ± SEM. Statistical significance was tested using one-way analysis of variance with post-hoc Tukey's tests. $^{***}P < 0.001$; ns, not significant.
